# Low fasting low high-density lipoprotein and postprandial lipemia

**DOI:** 10.1186/1476-511X-3-18

**Published:** 2004-07-23

**Authors:** Genovefa D Kolovou, Katherine K Anagnostopoulou, Nektarios Pilatis, Nikolaos Kafaltis, Konstandina Sorodila, Eleftherios Psarros, Dennis V Cokkinos

**Affiliations:** 1 1^st ^Cardiology Department, Onassis Cardiac Surgery Center, 17674, Athens, Greece; 2 Biochemistry Laboratory, Onassis Cardiac Surgery Center, 17674, Athens, Greece

**Keywords:** low high-density lipoprotein cholesterol, coronary heart disease, postprandial lipemia, triglyceride clearance.

## Abstract

**Background:**

Low levels of high density lipoprotein (HDL) cholesterol and disturbed postprandial lipemia are associated with coronary heart disease. In the present study, we evaluated the variation of triglyceride (TG) postprandially in respect to serum HDL cholesterol levels.

**Results:**

Fifty two Greek men were divided into 2 main groups: a) the low HDL group (HDL < 40 mg/dl), and b) the control group. Both groups were further matched according to fasting TG (matched-low HDL, and matched-control groups). The fasting TG concentrations were higher in the low HDL group compared to controls (*p *= 0.002). The low HDL group had significantly higher TG at 4, 6 and 8 h postprandially compared to the controls (*p *= 0.006, *p *= 0.002, and *p *< 0.001, respectively). The matched-low HDL group revealed higher TG only at 8 h postprandially (*p *= 0.017) compared to the matched-control group. ROC analysis showed that fasting TG ≥ 121 mg/dl have 100% sensitivity and 81% specificity for an abnormal TG response (auc = 0.962, *p *< 0.001).

**Conclusions:**

The delayed TG clearance postprandially seems to result in low HDL cholesterol even in subjects with low fasting TG. The fasting TG > 121 mg/dl are predictable for abnormal response to fatty meal.

## Background

The hypothesis that low levels of high density lipoprotein (HDL) cholesterol is associated with coronary heart disease (CHD), raised since the 1950s [1]. Fifty years later, it was well-established [2,3] as it has been excellently proved after a number of large prospective studies [4,5]. In the PROCAM Study [6] 45% of men and women who developed CHD had an HDL cholesterol lower than 35 mg/dl. In the Framingham Heart Study, total cholesterol levels did not provide a predictive value in identifying people at risk for CHD compared to cholesterol/HDL ratio [7]. Furthermore, a change in ratio is better predictor for successful CHD risk reduction than changes in total cholesterol levels. There is no longer any doubt that HDL cholesterol is a powerful independent inverse predictor of CHD. On the other hand, the long duration of the postprandial lipemia and repetition of meals during the daytime leads to important changes of lipoproteins postprandially. Studies have shown that disturbed postprandial lipemia is found in patients with CHD [8,9] and other conditions [10-12] related to an increased risk of cardiovascular disease. Many studies comparing patients with CHD and controls have shown that postprandial triglyceride (TG) levels were an independent predictor of CHD in multivariate analysis [8,13]. In the present study, we evaluated the variation of TG postprandially in respect to serum HDL cholesterol levels. The delayed TG clearance postprandially seems to result in low HDL cholesterol even in subjects with low fasting TG.

## Results

All participants ingested their fatty meal and tolerated it well.

### Baseline characteristics (Table 1)

**Table 1:** Clinical characteristics of the two main study groups (all low HDL patients and controls). All biochemical values were obtained in the fasting state and for TG 4,6,8 h postprandially.

Characteristics	Low HDL n = 29	Controls n = 23	P values
Age (years)	45(13)	51(9)	NS
BMI (kg/m^2^)	26(2)	26(3)	NS
CHD -/+	19/10	23/0	0.002
Hypertension-/+	27/2	23/0	NS
Smokers-/+	17/12	23/0	<0.001
Diabetes mellitus	28/1	23/0	NS
TC (mg/dl)	198(45)	194(38)	NS
HDL (mg/dl)	31(7)	53(19)	<0.001
LDL (mg/dl)	141(39)	124(38)	NS
Apo A (mg/dl)	123(28)	153(42)	0.007
Apo B (mg/dl)	127(56)	126(30)	NS
Lp (a) (mg/dl)	25(25)	30(29)	NS
Glucose (mg/dl)	94(13)	90(11)	NS
TG_0 _(mg/dl)	128(54)	89(30)	0.002
TG_4 _(mg/dl)	207(102)	140(36)	0.006
TG_6 _(mg/dl)	210(112)	135(45)	0.002
TG_8 _(mg/dl)	192(92)	103(37)	<0.001
AUC (mg/dl/h)	1459(631)	1007(253)	<0.005

All values are presented as means (standard deviation). BMI: body mass index, CHD: coronary heart disease, TC: total cholesterol, HDL: high-density lipoprotein cholesterol, LDL: low-density lipoprotein cholesterol, Apo: apolipoprotein, Lp: lipoprotein, TG_0_: fasting plasma triglyceride concentration, TG_4, _TG_6, _TG_8_: plasma triglyceride concentration 4,6,8 h after the fat load, respectively, AUC: area under the curve. For TC, HDL and LDL, to convert from mg/dl to mmol/L divide by 38.7 For TG, to convert from mg/dl to mmol/L divide by 88.6

The clinical characteristics of the two main study groups (low HDL and controls) are shown in Table 1. Renal and liver function was normal as determined by measuring plasma creatinine, urea, uric acid, alanine aminotransferase, aspartate aminotransferase and γ-glutamate transferase. Therefore, there was no biochemical evidence of a fatty liver. Baseline glucose levels were similar in both groups. None of the patients fulfilled the criteria for the metabolic syndrome according to the National Cholesterol Education Program- Adults Treatment Panel III (NCEP ATP III) guidelines [14].

The plasma HDL cholesterol and apolipoprotein A were lower in low HDL group compared to controls (*p *< 0.001, and *p *= 0.007, respectively) by definition. The fasting TG (TG_0_) concentrations were higher in low HDL cholesterol group compared to controls (*p *= 0.002).

### Postprandial TG concentrations in the two main groups (low HDL subjects and controls) (Table 1, Figure 1)

**Figure 1:**
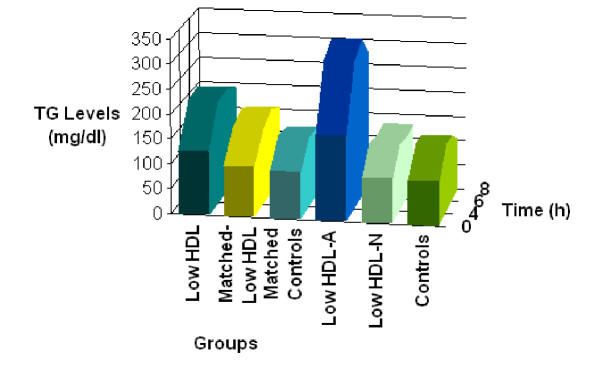
Schematic representation of TG-AUC of all groups in relation to time. The highest AUC values were observed in low HDL and low HDL-A groups which are characterized by low fasting HDL levels and the highest fasting TG levels compared to the other groups. The lowest AUC values were found in matched-low HDL, matched-controls, low HDL-N and control groups. These are characterized by lower fasting TG levels but with variable fasting HDL levels, ranging from 30(9) to 55(20) mg/dl. This means that the primary determinant of the magnitude of TG postprandial response is the fasting TG concentration. TG-AUC: triglyceride-area under the curve Low HDL-A: low HDL-Abnormal group Low HDL-N: low HDL-Normal group

#### TG levels 4 h after the fatty meal (TG_4_)

The low HDL subjects had a significantly higher (*p *< 0.006) TG level compared to the controls.

#### TG levels 6 h after the fatty meal (TG_6_)

The low HDL subjects had a significantly higher (*p *< 0.002) TG level compared to controls.

#### TG levels 8 h after the fatty meal (TG_8_)

The low HDL subjects had a significantly higher (*p *< 0.001) TG level compared to the controls.

Glucose did not show any change postprandially.

The above groups were subdivided in matched-low HDL and matched-control group (matched for low fasting TG levels) and in low HDL-Abnormal (low HDL-A) and low HDL-Normal (low HDL-N) groups (based on their TG postprandial response).

### Results of the matched low HDL subjects and matched controls (Table 2, Figure 1)

**Table 2:** Clinical characteristics and biochemical parameters of the low HDL subjects matched to controls for fasting TG levels, and their response to a fatty meal.

Characteristics	Matched-low HDL n = 20	Matched-controls n = 20	P values
Age (years)	47(13)	51(10)	NS
BMI (kg/m^2^)	26(2)	25(2)	NS
CHD -/+	11/9	20/0	0.001
Hypertension-/+	18/2	20/0	NS
Smokers-/+	13/7	20/0	0.004
TC (mg/dl)	187(50)	197(35)	NS
HDL (mg/dl	31(8)	55(20)	<0.001
LDL (mg/dl)	136(43)	128(38)	NS
Apo A (mg/dl)	123(31)	158(43)	0.009
Apo B (mg/dl)	123(63)	132(27)	NS
Lp (a) (mg/dl)	29(27)	28(23)	NS
Glucose (mg/dl)	95(14)	90(11)	NS
TG_0 _(mg/dl)	100(31)	95(27)	NS
TG_4 _(mg/dl)	153(55)	140(36)	NS
TG_6 _(mg/dl)	172(105)	138(45)	NS
TG_8 _(mg/dl)	154(78)	105(38)	0.017
AUC (mg/dl/h)	1142(322)	1007(253)	NS

All values are presented as means (standard deviation). BMI: body mass index, CHD: coronary heart disease, TC: total cholesterol, HDL: high-density lipoprotein cholesterol, LDL: low-density lipoprotein cholesterol, Apo: apolipoprotein, Lp: lipoprotein, TG_0_: fasting plasma triglyceride concentration, TG_4, _TG_6, _TG_8_: plasma triglyceride concentration 4,6,8 h after the fat load, respectively, AUC: area under the curve. For TC, HDL and LDL, to convert from mg/dl to mmol/L divide by 38.7 For TG, to convert from mg/dl to mmol/L divide by 88.6

Subjects with low HDL cholesterol had higher TG concentration at 8 h postprandially (*p *= 0.017).

### Results of the low HDL subjects divided into those with (low HDL-N group) and without (low HDL-A group) a normal response to a fatty meal (Table 3, Figure 1)

**Table 3:** Clinical characteristics and biochemical parameters of the low HDL subjects with an abnormal (low HDL-A) and normal response (low HDL-N) to a fatty meal.

Characteristics	low HDL-A n = 13	low HDL-N n = 16	P values
Age (years)	42(14)	48(13)	NS
BMI (kg/m^2^)	26(2)	27(3)	NS
CHD-/+	12/1	7/9	0.006
Hypertension-/+	11/2	16/0	NS
Smokers-/+	6/7	11/5	NS
TC (mg/dl)	221(16)	179(53)	0.007
HDL (mg/dl)	32(5)	30(9)	NS
LDL (mg/dl)	154(22)	130(46)	NS
Apo A (mg/dl)	131(18)	118(31)	NS
Apo B (mg/dl)	135(17)	122(68)	NS
Lp (a) (mg/dl)	10(7)	32(28)	0.012
Glucose (mg/dl)	92(11)	95(14)	NS
TG_0 _(mg/dl)	172(43)	92(29)	<0.001
TG_4 _(mg/dl)	298(82)	135(35)	<0.001
TG_6 _(mg/dl)	321(74)	127(35)	<0.001
TG_8 _(mg/dl)	270(78)	129(37)	< 0.001
AUC (mg/dl/h)	2074(381)	986(252)	<0.012

All values are presented as means (standard deviation) BMI: body mass index, CHD: coronary heart disease, TC: total cholesterol, HDL: high-density lipoprotein cholesterol, LDL: low-density lipoprotein cholesterol, Apo: apolipoprotein, Lp: lipoprotein, TG_0_: fasting plasma triglyceride concentration, TG_4, _TG_6, _TG_8_: plasma triglyceride concentration 4,6,8 h after the fat load, respectively, AUC: area under the curve. For TC, HDL and LDL, to convert from mg/dl to mmol/L divide by 38.7 For TG, to convert from mg/dl to mmol/L divide by 88.6

Thirteen (45%) of the low HDL subjects (low HDL-A group) had an abnormal TG response to the fatty meal test compared to the controls. The other sixteen low HDL subjects (low HDL-N group) had a TG response to the fatty meal that was similar to that in the control subjects. The low HDL subjects with an abnormal fatty meal response (low HDL-A group) had higher plasma total cholesterol and lower lipoprotein (a) concentrations (*p *= 0.007, and *p *< 0.012, respectively). As expected, postprandial TG levels were higher in those classified as having an abnormal response.

TG levels of all groups (low HDL, controls, matched low HDL, matched controls, low HDL-A, low HDL-N) in relation to time are schematically represented in Figure 1.

### Predictors of abnormal TG response

In multivariate linear regression analysis, where the independent variables were age, body mass index (BMI), total cholesterol, HDL cholesterol, TG, lipoprotein (a), and areas under the curve (AUC) was the dependent variable, the TG_0 _was the only predictor of high AUC values (Coefficience B = 7.15, *p *= 0.016). ROC analysis showed that TG_0 _levels ≥ 121 mg/dl have 100% sensitivity and 81% specificity for an abnormal TG response (auc=0.962, *p *< 0.001). After we divided the low HDL men in low- or high- groups by using the ROC curve cut-off values of TG_0_, total cholesterol and lipoprotein (a), i.e. low/high TG_0_, low/high total cholesterol and low/high lipoprotein (a) groups, the only distinction that predicted an abnormal TG response was the high-TG_0 _group (*p *< 0.001)

## Discussion

In the present study, we found that a slower clearance of TG-rich lipoproteins from circulation postprandially results in low fasting levels of HDL cholesterol. Additionally, fasting plasma TG concentration is the primary determinant of the magnitude of postprandial lipemia.

Subjects with a low fasting HDL and low TG levels showed a delayed TG clearance at 8 h compared to those with a normal fasting HDL value. The increase in TGs (from 2 to 4 h) after meal consumption mainly reflects dietary TG absorption, whereas the return to fasting levels (from 6 to 9 h) is presumably a function of TG clearance [15]. In our study, the delayed response of TGs to the fatty meal in matched group for low fasting TG levels may be indicative for an HDL involvement. It has been proposed that elevated plasma TG concentrations promote the cholesterol ester exchange reactions mediated by cholesteryl ester transfer protein [16]. It is possible that in this transient hypertriglyceridemia, the HDL particles are TG-enriched via cholesteryl ester transfer protein mediated exchange with TG-rich lipoproteins. Such HDL-TG enriched particles are cleared more rapidly from the circulation [17] leading to low serum HDL cholesterol levels [18,19]. In subjects with initially low HDL concentrations and low fasting TG levels, it is possible that this reaction does not happen at all or happens in a less degree and the slower removal of TGs from the circulation could be explained by insufficient amount of HDL particles which are responsible for their clearance.

The high TG and low HDL cholesterol phenotype has been frequently reported in "abdominal" obesity [20,21]. Obesity is associated with a range of metabolic abnormalities including fasting and postprandial dyslipidemia. This was clearly shown in the study of Couillard et al., whose study subjects were characterised by a BMI of 32.3 ± 4.5 kg/m^2 ^[22]. Our results showed that even in slightly overweight subjects with a BMI of 26 ± 2 kg/m^2^, this phenotype may provoke similar metabolic abnormalities. The HDL formation is closely associated with TG catabolism [23] as mentioned above. The deleterious effect of the high TG and low HDL phenotype on the rate of postprandial TG clearance has been also shown in children (mean age 14 years old) [24]. Patsch et al., [25] have proposed that low HDL cholesterol levels could result from an impaired TG lipolysis, a condition that would favour an exaggerated postprandial lipemia, in subject with hypoalphalipoproteinemia.

In our study, no difference was observed in the early postprandial TG response (absorption phase) between normolipidemic controls and men with low fasting HDL cholesterol and low fasting plasma TG concentration. In contrast, the group with low HDL cholesterol (31 ± 7 mg/dl) and variable values of fasting TG (53–248 mg/dl, mean value 128 ± 54 mg/dl) revealed a higher TG curve postprandially compared to healthy subjects with moderate HDL cholesterol (53 ± 19 mg/dl) and low fasting TG levels (89 ± 30 mg/dl). When both groups were matched for fasting TG levels, this curve-difference was diminished, although HDL cholesterol levels remained significantly different between the two groups. When subjects with low HDL cholesterol were subdivided based on their response (normal or abnormal) to fat loading, fasting TG concentration seemed to be the critical determinant of such response. Since there are no official guidelines to determine normal postprandial TG ranges, the characterization of a TG response as "normal" or "abnormal" after fat loading was based on this report and on previous reports of ours and others. The peak TG mean value in control subjects was 139 ± 41 mg/dl (at 6 h) [11], 176 ± 17 mg/dl (at 3.5 h) [26], 177 mg/dl [27] and 186 mg/dl [28]. Higher TG concentrations at 4 h were reported by others, approximately 213 mg/dl and 248 mg/dl [29-32]. Therefore, we defined an abnormal postprandial TG response to the fatty meal as any postprandial TG concentration (at 4, 6 or 8 h) higher than 219 mg/dl which was the highest TG concentration in any hour in any control individual. Subjects with abnormal postprandial lipemia had fasting TG concentration twice higher than those with normal postprandial lipemia and the TG concentration remained much higher through all tests. Additionally, the ROC analysis showed that TG_0 _level ≥ 121 mg/dl have 100% sensitivity and 81% specificity for an abnormal TG response.

Couillard et al., [22] reported a significant association between the magnitude of the postprandial TG response and fasting plasma HDL cholesterol concentrations. However, the subjects included in their study showed a wider range of fasting TG concentrations (44–390 mg/dl) compared to ours (53–248 mg/dl). In the subgroup of isolated low fasting HDL cholesterol, fasting hypertriglyceridemia was a prerequisite in order to have an exaggerated postprandial TG response [22]. This finding was similar to ours; an abnormal response to the fatty meal was only dependent on fasting TG concentration. Cohen and co-workers [33] measured plasma TG and retinyl palmitate responses to different fat meals in endurance-trained men with a wide range of plasma HDL cholesterol concentrations (36–105 mg/dl). Their data indicated that the magnitude of postprandial lipemia is not primarily affected by the HDL cholesterol concentrations, which agrees to our results. However, they failed to show any correlation between HDL cholesterol levels and chylomicron remnant metabolism which is contradictory to our findings, although we did not directly measured chylomicron remnant particles.

Postprandial hypertriglyceridemia is not a uniform abnormality. It pathophysiologic cause is not yet known. It is possible that the response to a fatty meal is gene dependent. It has been reported that a number of gene loci, such as these of apolipoprotein E, lipoprotein lipase, apolipoprotein CIII, apolipoprotein A1, apolipoprotein A4, cholesterol ester transfer protein are related to fat load response [34,35]. However, this gene polymorphism-dependence remains controversial, and it is more likely that the postprandial lipemia is a polygenic phenomenon, although the phenotype of the postprandial lipemia is probably one. This concept allowed us to study only the phenotypic manifestation of the postprandial lipemia, which would have immediate clinical implications. Our aim was to evaluate the postprandial response in men with isolated low fasting HDL cholesterol. Although it has been also described elsewhere that baseline TG levels impact on the postprandial response and that HDL cholesterol levels are predictors of this response, the novelty of this study lies on the fact that delayed postprandially TG clearance was observed in low HDL group and that emphasis should be given on the decrease of TG levels < 121 mg/dl, lower that those indicated by the NCEP ATP III guidelines.

## Conclusions

The delayed TG clearance postprandially seems to result in low HDL cholesterol levels even in subjects with low fasting TG concentration. Moreover, fasting plasma TG levels appear to be the primary determinant of the magnitude of postprandial lipemia. The fasting TG levels higher than 121 mg/dl are predictable for abnormal response to a fatty meal. This should be taken into account for the management of patients with fasting low HDL cholesterol levels.

## Methods

### Subjects

The study population consisted of 52 Greek men recruited from the Lipid Clinic of Onassis Cardiac Surgery Center, Athens, Greece. Heavy drinking, liver and renal disease, diabetes mellitus, metabolic syndrome, according to the NCEP ATP III guidelines [14], hypothyroidism and professional sport activity were exclusion criteria. No subject took lipid lowering drugs before entering the study. Only patients with CHD (defined by angiography) were taking soluble aspirin (100 mg) and isosorbide mononitrate (40 mg). No other patient or control subject was on medication.

The study population was divided into 2 main categories:

A: The low HDL group and the control group.

1. The low HDL group consisted of 29 men, mean age 45(13) years with low HDL cholesterol (< 40 mg/dl) according to NCEP ATP III guidelines [14].

**2. **The control group consisted of 23 healthy men, mean age 51(9) years with no family history of premature atherosclerosis, diabetes mellitus, arterial hypertension or dyslipidemia. Their fasting TG levels were < 150 mg/dl, total cholesterol < 240 mg/dl and HDL cholesterol = 40 mg/dl. All subjects were never smokers.

B: Matched groups according to fasting TG level.

1. The matched-low HDL group consisted of 20 men, mean age 47(13) years with low HDL cholesterol and low fasting TG levels [100(31) mg/dl].

2. The matched controls consisted of 20 men, mean age 51(10) years with lasting TG levels 95(27) mg/dl.

### Fat-rich meal protocol and blood sample

All patients were studied in the outpatient clinic between 8.00–9.00 am after 12 hours (h) overnight fast. The fatty meal was consumed within 20 min and plasma TG concentrations were measured before and 4, 6 and 8 h after the fat load. During this 8 h period, the participants did not eat; they could only drink water and they did not smoke. Blood samples were drawn at 8:00 am (before the meal), at 12:30 pm (4 h after the meal), at 2:30 pm (6 h after the meal), and at 4:30 pm (8 h after the meal). In all samples total cholesterol, TG, HDL, apolipoprotein A and B, and lipoprotein (a) were measured. BMI was calculated as weight divided by height (expressed in kg/m^2^).

#### Fatty meal

The fatty meal has been previously described [11]. Briefly, the fatty meal was a slight modification of that described by Patsch et al [8], consisting of 83.5% fat, 14.0 % carbohydrates and 2.5 % proteins. This was administered in a dose based on the patient's body surface area (350 g to 2 m^2^).

#### Definition of abnormal postprandial response

We defined postprandial hypertri-glyceridemia as any postprandial TG concentration (at 4, 6 or 8 h) higher than the highest TG concentration in any hour in any control individual; this TG value was 219 mg/dl.

#### Determination of blood lipids and glucose

Plasma total cholesterol, TG and HDL cholesterol were measured using enzymatic colorimetric methods on a Roche Integra Biochemical analyzer with commercially available kits (Roche). The serum low-density lipoprotein cholesterol levels were calculated using the Friedewald formula [36]. Apolipoprotein A, B and Lipoprotein (a) were measured by nephelometery (Nephelometer:BN-100, Behring, Germany). Blood glucose was measured by the hexokinase method with a Dade Behring reagent on a Dimension (Dade Behring) instrument. All samples were analyzed within 24 h.

All participants gave their informed consent before the study. The ethical committee of the Onassis Cardiac Surgery Center approved the study.

### Statistical analysis

Values of numerical characteristics were tested for normality and are presented as mean value with one standard deviation, when normally distributed, or as median and range, when they deviated from normality. A t-test for independent samples or a Mann Whitney U test was used for the comparison of numerical values between two groups and ANOVA or Kruskal-Wallis test for three groups, with Bonferoni correction for post hoc analysis. The comparison of clinical categorical variables was performed with the use of chi-square test. Multivariate linear regression analysis was performed to determine the predictors of elevated AUC levels, where age, BMI, total cholesterol, HDL cholesterol, fasting TG (TG_0_), and lipoprotein (a) were the independent variables tested. ROC curve statistics was used to estimate the cutoff value of baseline triglyceride, cholesterol and lipoprotein (a) levels (which they were significant univariate predictors of elevated TG levels postprandially) above which the low HDL men develop an abnormal TG response to fat load. Using those cut-off values, we created dichotomous variables for each one of the univariate predictors to test their significance in discriminating patients with abnormal TG response to fat load. The level of significance was set at *p *< 0.05. AUC for serial measurements of TG levels at baseline and after the fatty meal were calculated using the trapezoid rule.

## Authors' contributions

GK conceived the study and participated in the development of the hypothesis, the study design and drafting of the manuscript.

KA is a research associate who participated in the development of the hypothesis, study design and drafting of the manuscript.

NP participated in data analysis and in the interpretation of the findings.

NK is a physician who participated in the study design and recruitment of subjects, clinical evaluation and collection of blood samples.

KS is a senior nurse who participated in the study design, in the recruitment of subjects and in blood sample collection.

EP is a biochemist who participated in the laboratory part of the study.

DC participated in the design of the study and its coordination.
